# Exercise intolerance in pulmonary hypertension: robbing Peter to pay Paul

**DOI:** 10.1113/JP288081

**Published:** 2024-12-09

**Authors:** Ken D. O'Halloran

**Affiliations:** ^1^ Department of Physiology University College Cork Cork Ireland

**Keywords:** diaphragm dysfunction, pulmonary hypertension, vascular dysfunction

 

*Laboured debt, so we must borrow*

*Paid today, but not tomorrow*.

Cost of Living

The diaphragm is a remarkable striated muscle. As the principal muscle of inspiration, it is cyclically active facilitating airflow into the lungs from the first breath that we draw to our last. High force‐dependent actions such as airway clearance and cough require powerful contractions of the diaphragm. Moreover, the diaphragm is critical to the development of raised intra‐abdominal pressures subserving a range of functions. Notwithstanding its critical importance, the diaphragm is inherently vulnerable. Structural and functional plasticity, as seen in all striated muscle, affords advantages in the face of stressors but it can also provide a substrate for aberrant remodelling in circumstances that drive maladaptation. It is established that clinically relevant diaphragm weakness emerges in a wide range of scenarios such as critical care, muscle wasting diseases, undernutrition and endocrine disorders. It is also established that diaphragm weakness presents in both systemic and pulmonary hypertension.

Pulmonary hypertension (PH) is a progressive disease associated with limited exercise capacity and dyspnoea, relating to cardiac dysfunction, and peripheral and inspiratory muscle dysfunction. Humans and rats with PH show evidence of diaphragm muscle atrophy and weakness before the development of limb muscle dysfunction. PH‐induced diaphragm weakness is partly attributable to sarcomeric dysfunction (Manders et al., 2012), which may relate to redox stress amenable to rescue by chronic antioxidant treatment (Himori et al., 2017). Maximum inspiratory pressure is reduced in people with PH, with evidence of decreased force in permeabilised diaphragm slow fibres and decreased calcium sensitivity in fast fibres, rescued by a fast troponin activator (Manders et al., 2016).

The work of breathing is increased in PH, necessitating greater blood flow to meet increased oxygen demand. PH impairs vasomotor function in diaphragm arterioles, alters blood flow distribution and impairs the hyperaemic response to electrically induced contractions of the *in situ* diaphragm (Schulze et al., 2023). These findings suggest that vascular dysfunction may precede and contribute to diaphragm dysfunction in PH due to a mismatch in oxygen delivery and demand.

In this issue of *The Journal of Physiology*, Schulze et al. (2024) extend their recent work to assess limb and respiratory muscle blood flow during aerobic exercise in rats with PH to determine the relative distribution of cardiac output to working muscles in PH, and in the light of previous findings (Schulze et al., 2023), assess bulk and regional blood flow distribution in the diaphragm during dynamic sub‐maximal exercise. In essence, the study sought to determine if altered haemodynamics between and within muscles predicates exercise intolerance in PH.

Studies were performed in control and monocrotaline‐induced PH rats. Echocardiography confirmed disease progression with experimental studies performed in rats with moderate PH, preceding right heart failure. Maximal oxygen uptake and exercise performance were lower in PH rats. Blood flow (quantified by established methods using fluorescent microspheres) during exercise at the same absolute workload was greater in the diaphragm and lower in the soleus muscle of the limb in PH rats compared to control rats. This demonstrates a potentiation in PH rats of the respiratory muscle ‘raid’ on cardiac output to pay the debt of increased workload, which limits exercise tolerance. Performance is curtailed owing to decreased blood flow to locomotor muscles working at a higher relative workload. Interestingly, bulk diaphragm blood flow during exercise increased to a similar extent in PH rats as in control rats, despite prior evidence of impaired vasomotor and blunted hyperaemic responses in PH diaphragm (Schulze et al., 2023). This strongly suggests heterogeneity in vascular remodelling in PH and a distribution of the inspiratory burden to other compartments of the muscle as evidenced by increased medial costal and ventral costal blood flow. The higher demand on respiratory muscles in PH was further evidenced by increased intercostal blood flow during exercise, which was seen only in PH rats.

Reasonably, the authors conclude that exercise tolerance in PH is limited by an exaggerated raid by the respiratory muscles on cardiac output at the expense of blood flow to locomotor muscles. This is framed by Schulze et al. (2024) as a pathological ‘steal’ by the diaphragm with regional distribution of the bounty to potentially less mechanically efficient compartments of the muscle and a greater reliance on other obligatory muscles such as the intercostals. Increased reliance on regional and extra‐diaphragmatic compartments in response to increased workload may be adaptive (albeit at the expense of locomotor muscle blood supply) or may be driven by pathophysiological changes in diaphragm vascular function and/or control. Further work is required to determine the temporospatial and inter‐dependent relationship between vascular and muscle remodelling in the PH diaphragm.

PH was induced in rats by single injection of monocrotaline, a widely used model, which induces pulmonary endothelial damage and vascular remodelling. Whereas systemic vascular damage is unlikely (and at the dose used was not previously seen in liver, spleen or kidney), a direct effect of monocrotaline on diaphragm vasculature, independent of PH, cannot be excluded. Expansion of the current work to other models of PH, such as chronic hypoxia, is on the face of things warranted. However, such studies are problematic as chronic hypoxia causes respiratory and limb muscle atrophy and intrinsic myofibre weakness, although this may be dependent upon or aggravated by PH. Nevertheless, since exposure to hypoxia for just 8 h (without PH) causes diaphragm dysfunction (O'Leary et al., 2018), it is evident that hypoxia is a confounder, and yet local hypoxia may be a critical factor in PH.

The work of Schulze et al. (2024) is a reminder of the remarkable capacity for physiological adjustments to stress (adaptation), requiring trade‐offs, with the cost of the cumulative burden inevitably leading to a spiral of disability common in disease states (maladaptation). Strategies to subserve function in one domain come at a cost to performance in another. In this way, the haemodynamic conundrum in PH is a case of robbing Peter to pay Paul. Manoeuvring the Apostles works (increased respiratory work in the quest for adequate oxygenation, at least at rest) until it does not (dyspnoea and exercise intolerance). Importantly, the new findings suggest that targeting the diaphragm vasculature offers a potential therapeutic strategy for people with PH.

## Additional information

### Competing interests

None declared.

### Author contributions

Ken O'Halloran: Conception or design of the work; Drafting the work or revising it critically for important intellectual content; Final approval of the version to be published; Agreement to be accountable for all aspects of the work.

### Funding

None.

## Supporting information


Peer Review History
